# Mesenchymal Stem Cells: A New Piece in the Puzzle of COVID-19 Treatment

**DOI:** 10.3389/fimmu.2020.01563

**Published:** 2020-07-03

**Authors:** Felipe Saldanha-Araujo, Emãnuella Melgaço Garcez, Amandda Evelin Silva-Carvalho, Juliana Lott Carvalho

**Affiliations:** ^1^Hematology and Stem Cells Laboratory, Health Sciences Department, University of Brasília, Brasilia, Brazil; ^2^Molecular Pharmacology Laboratory, Health Sciences Department, University of Brasília, Brasilia, Brazil; ^3^Multidisciplinary Laboratory of Biosciences, Faculty of Medicine, University of Brasilia, Brasilia, Brazil; ^4^Genomic Sciences and Biotechnology Program, Catholic University of Brasília, Brasilia, Brazil

**Keywords:** COVID-19, mesenchymal stem cells, stem cell-therapy, SARS-CoV-2, clinical trials

## Abstract

COVID-19 is a disease characterized by a strong inflammatory response in severe cases, which fails to respond to corticosteroid therapy. In the context of the current COVID-19 outbreak and the critical information gaps regarding the disease, several different therapeutic strategies are under investigation, including the use of stem cells. In the present manuscript, we provide an analysis of the rationale underlying the application of stem cells to manage COVID-19, and also a comprehensive compendium of the 69 clinical trials underway worldwide aiming to investigate the application of stem cells to treat COVID-19. Even though data are still scarce, it is already possible to observe the protagonism of China in testing mesenchymal stem cells (MSCs) for COVID-19. Furthermore, it is possible to determine that current efforts focus on the use of multiple infusions of high numbers of stem cells and derived products, as well as to acknowledge the positive results obtained by independent groups who publicized the therapeutic benefits provided by such therapies in 51 COVID-19 patients. In such a rapid-paced field, up-to-date systematic studies and meta-analysis will aid the scientific community to separate hype from hope and offer an unbiased position to the society and governments.

## Introduction

In December 2019, the world witnessed the first reports of patients presenting pneumonia in the city of Wuhan, China (1). Soon after, genome sequencing studies demonstrated that such cases were associated with a new coronavirus, named Severe Acute Respiratory Syndrome CoronaVirus 2 (SARS-CoV-2). The disease caused by such a virus was named coronavirus disease 2019, or simply COVID-19 (2).

The COVID-19 pandemic has taken aback governments, health systems, and also scientists. Still, the formidable challenges of understanding the biology of SARS-CoV-2 and the pathophysiology of COVID-19, in order to develop effective treatment and immunization protocols, have led to a fast worldwide mobilization of the scientific community. Countless studies and position articles have been published regarding COVID-19 and are set to expand rapidly.

Stem cells constitute important assets for animal-free experiments, providing cheaper, and ethical alternatives to animal studies. In this sense, scientific papers have already described the application of stem cells to generate new data regarding several aspects of SARS-CoV-2 (3), similar to what has been done with other viral diseases in the past (4). In addition to the possible application of stem cells in basic research, such cells are also appealing tools for immunomodulation, as shown by our group and others (5–12), as well as tissue regeneration, with a great deal of accumulated knowledge regarding the underlying mechanisms of action and decades of clinical experience, as shown by our group and others (13–18). It is well-established that Mesenchymal stem cells (MSCs) are able to control the functions of most if not all immune cells, and that such effects occur through a network of mechanisms including direct cell-cell contact, and secretion of soluble factors with immunosuppressive function (19, 20). MSCs may therefore be promising tools for the management of disorders involving immune system dysregulation, which may be the case of COVID-19.

In the context of the world-wide COVID-19 pandemic, clinical trials have been initiated, even though much necessary information regarding the disease is still lacking. Over 1,700 clinical studies have been registered worldwide, focusing on investigating COVID-19 and trying to validate novel therapeutic regimens, immunization protocols, and anti-viral strategies (World Health Organization International Clinical Trials Registry Platform). Among them, 69 involve the use of stem cells and stem cell-derived products.

In the present manuscript, we provide an up-to-date analysis of the rationale and current studies involving the clinical application of stem cells to manage COVID-19. Even though data are still scarce, it is already possible to determine the state of the art, the main groups dedicating efforts in the theme, and also the short-term perspectives for COVID-19 management using stem cells.

## Covid-19 Pathophysiology and the Rationale for Using Stem Cells

Coronaviruses were first isolated from patients with common cold by Tyrrell and Bynoe in (21). They constitute enveloped, positive-sense RNA viruses, capable of infecting various mammals, including humans (21). The SARS-CoV-2 belongs to the *Nidovirales* order, *Coronaviridae* family, *Coronavirinae* subfamily. The *Coronavirinae* is further divided into the alpha, beta, gamma, and delta coronaviruses. While coronaviruses alpha and beta seem to have originated from mammals (especially bats), the gamma and delta coronaviruses seem to derive from pigs and birds (22). Prior to the present SARS-CoV-2 outbreak, coronaviruses were only thought to cause mild infections in humans. Now it is considered that among the seven coronaviruses subtypes that are capable of infecting humans, the beta coronaviruses–including SARS-CoV-2 -, may cause severe and fatal diseases. SARS-CoV-2 spreads mainly through the respiratory airways through droplets that are shed from the respiratory secretions of infected persons, but also by direct personal contact (23). Recently, the SARS-Cov-2 has been isolated from fecal samples of severe pneumonia patients, suggesting other transmission routes (24).

**Figure 1:**
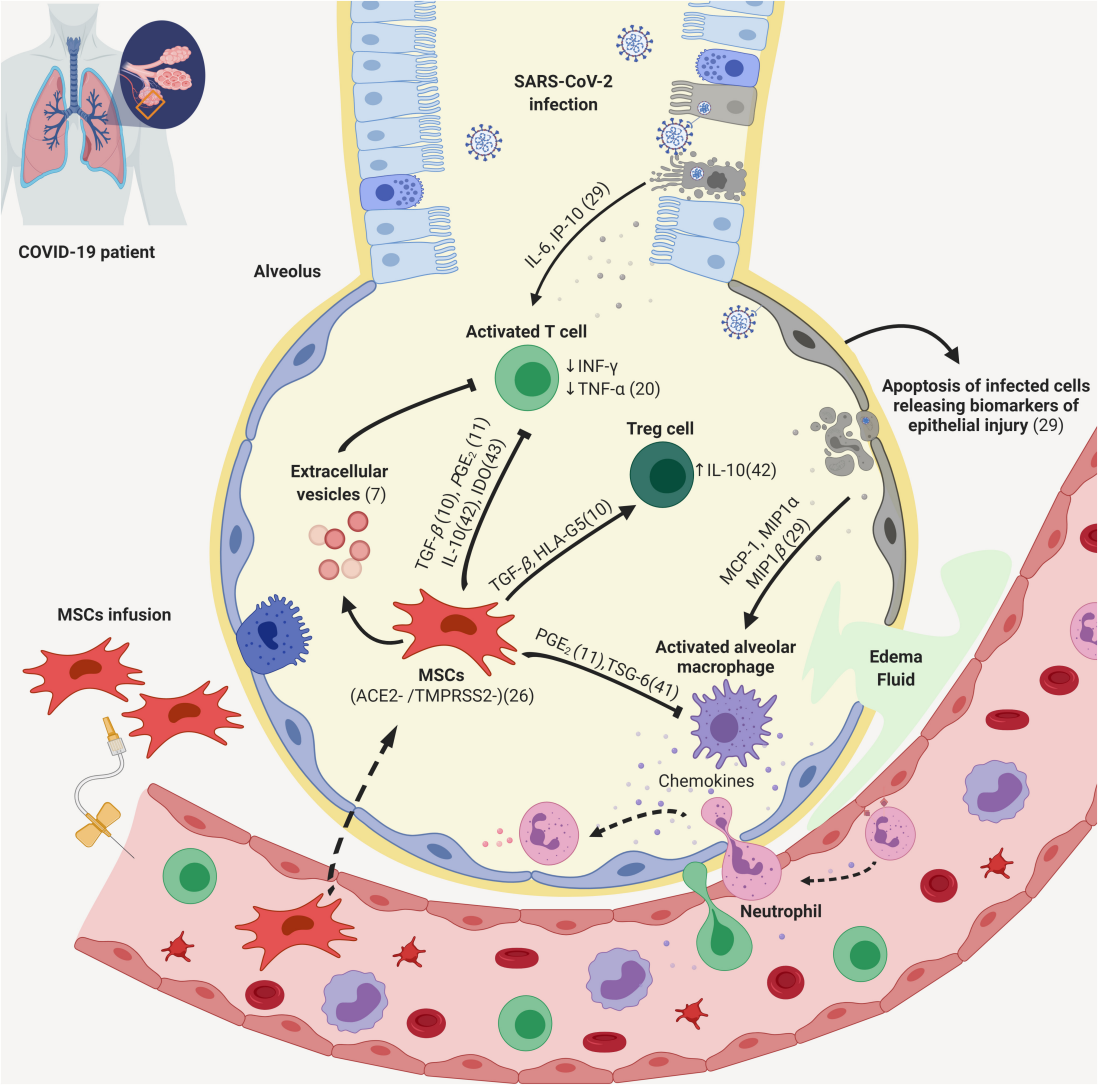
Putative mechanisms of action of MSCs against lung inflammation caused by COVID-19. During SARS-CoV-2 infection, lung ACE-2 positive cells, such as epithelial and capillary endothelial cells are infected, leading to cell damage, inflammatory signaling, and the release of cytokines and chemokines. The inflammatory *milieu* promotes the activation of local macrophages, dendritic, and endothelial cells, which further secrete soluble factors and promote the migration of circulating monocytes, granulocytes, as well as lymphocytes. This leads to a feed-forward process, characterized by inflammation, tissue destruction, and organ dysfunction. MSCs and their secretome can be used to counteract inflammation through contact-dependent and also paracrine processes. MSCs, Mesenchymal Stem Cells; Tregs, Regulatory T-cells. Created with BioRender.com.

It has been shown that SARS-Cov-2 is capable of infecting angiotensin I converting enzyme 2 (ACE-2) receptor-positive cells. Such a receptor is expressed by a wide variety of cell types, including the lung epithelial and capillary endothelial cells, in which it successfully replicates. Inflammatory cells were shown to be potentially infected by SARS-CoV viruses, but only leading to abortive infection (25). Still, viral entry engenders both innate and adaptive immune response, which initiate *in loco*, but soon are detectable in the serum of COVID-19 patients (26). The local tissue alterations provoked by the virus infection are characterized by intense inflammation, inflammatory cell migration, and edema (Figure 1). The subsequent tissue destruction and dysfunction are proportional to viral load and ACE-2 expression, which is increased in patients under Angiotensin-converting enzyme inhibitors /angiotensin receptor blockers therapy (27–29). Local tissue damage engendered by the viral infection is detectable by radiological analysis, even before the patient presents pneumonia symptoms.

COVID-19 can also affect the heart, liver, kidneys and alter the immune system (30), presenting a wide range of clinical outcomes that range from mild to common, severe, and critically severe states (26). In the latter scenario, patients require advanced life support, which is of great concern to public health systems (31).

The inflammatory response caused by SARS-Cov-2 is both the primary mechanism of viral elimination, but also tissue destruction and dysfunction (Figure 1). The internalization of the virus in tissue and immune cells leads to activation of nuclear factor-kappa B (NF-kB) pathway and secretion of a myriad of inflammatory factors, including IL-1, IL-17, TNF-α, and INF-γ (32–34). Hyperinflammation and cytokine storm, both of which can promote multiple organ failure, have been observed in severe and critically severe patients (35) justifying current efforts to test the therapeutic benefit of anti-inflammatory interventions, including corticosteroids, immunosuppressors, and inhibitors of Janus kinase (36).

Mesenchymal Stem Cells (MSCs) can be obtained from different tissues and are characterized by regenerative and immunomodulatory properties, which render them exciting tools for cell therapy. As reviewed by our group and others (10, 37, 38), MSCs present remarkable angiogenic, healing, antiapoptotic, and immunomodulatory potential. Furthermore, due to the low expression of MHC-I, MHC-II, and costimulatory molecules, they can be generally recognized as immune evasive and safe when used in allogeneic settings (39).

The immunomodulatory mechanisms of MSCs include cell contact-dependent (40) and paracrine processes, including the secretion of TNF-Stimulated Gene-6 (41), IL-10 (42), indoleamine 2,3-dioxygenase (43), adenosine (9), and also extracellular vesicles (7). Such processes lead to lower immune cell maturation and activation, in addition to promoting the differentiation of T-cells into regulatory T-cells (Tregs) (38).

Over 1,000 clinical studies have been performed up to date investigating the therapeutic potential of MSCs for various purposes, including diseases in which the immune system response is exacerbated, such as rheumatoid arthritis (44), Crohn's disease (45), Systemic lupus erythematosus (46–49) and graft- vs. -host disease (50). Despite the fact that many of such trials are still in progress, the available data obtained during the last 30 years clearly show that MSCs constitute promising tools in the treatment of inflammatory diseases. Considering inflammatory diseases, most consistent data relate to the use of MSCs in the treatment of the graft-vs. -host disease, highlighting the potential of MSCs to improve clinical signs of inflammation and favoring patient survival (50–53). Similar observations have been made in the context of other inflammatory disorders. For instance, it has been reported that MSCs infusions promote a reduction of the inflammatory status and ameliorates the clinical signs of patients with rheumatoid arthritis (54, 55). In Crohn's disease patients, several published studies revealed that MSCs induce perianal fistula healing (56–59). In recognition of the cited and other studies, FDA and EMA have conceded commercial approval for some MSC-based products targeting specific indications (e.g., Alofisel and Remestemcel-L, which are indicated to treat anal fistulas in Crohn's disease and graft- vs. -host disease, respectively).

Frequently, the treatment with MSCs follows intravenous administration of the cells. Interestingly, it has been shown that in this scenario a significant percentage of MSCs are rapidly trapped in the lungs upon intravenous injection, and also that lesioned sites increase MSC migration and retention (60). In the lung capillaries, the same occurs: upon injury, the local production of the pro-inflammatory mediator Angiotensin II (Ang II) is increased, and drives MSC migration *in vivo* through interactions with the Angiotensin II type 2 receptor (61–63). When trapped in the lungs, MSCs show the same immunomodulatory behavior as described in other body sites, such as the capacity of releasing anti-inflammatory cytokines (64), and antimicrobial peptides (65) (Figure 1).

As revised by Khoury et al. (66), at least 6 preclinical studies have been executed in order to investigate the therapeutic effects of MSCs in rodent (5 studies) and porcine (1 study) models of influenza virus infection. Two out of six studies described a lack of efficacy of MSC treatment in such models, but the most recent investigations (4 out of 6) have revealed positive outcomes, such as survival rate and decreased virus-induced inflammation. The capacity of influenza viruses to infect MSCs (which has not been assessed *in vivo*), host age, the varied MSC sources (umbilical cord and bone marrow), the different cell doses and administration routes involved in the studies may all help explaining the dissonant observations among research groups.

Clinically, the anti-inflammatory effects of MSCs have also been investigated in the context of Acute Respiratory Distress Syndrome (ARDS) with different etiologies (67–69). In a more recent study, perhaps as a hint for the application of MSCs for COVID-19 management, Chen et al. have infused MSCs into influenza A (H7N9) patients and obtained a significant reduction in mortality, compared to control group (70). In the cited study, 17 patients with ARDS caused by H7N9 infection were treated with menstrual blood-derived MSCs. While the control group (treated with antiviral agents, oxygen inhalation, etc) presented 54.5% mortality, only 17.6% of MSC-treated patients expired. Interestingly, 4 patients were followed during 5 years, with no harmful effects during this period.

Overall, the achieved results of MSC therapy for ARDS are promising, but important information is lacking in the literature, such as details on the management of control subjects, the time between MSC infusions, details regarding registered deaths, as well as other outcomes of interest, such as ventilator-free days and ICU stay.

Therefore, considering the acquired experience of the scientific and medical communities regarding the clinical application of MSCs to modulate the immune response, as well as the limited–but existent–pieces of evidence regarding the safety and efficacy of MSC therapy for viral respiratory infections, MSCs have entered clinical investigation for COVID-19 treatment. The current state of the art is reviewed below.

## Current Efforts to Treat Covid-19 Based on Stem Cell Products

**Figure 2:**
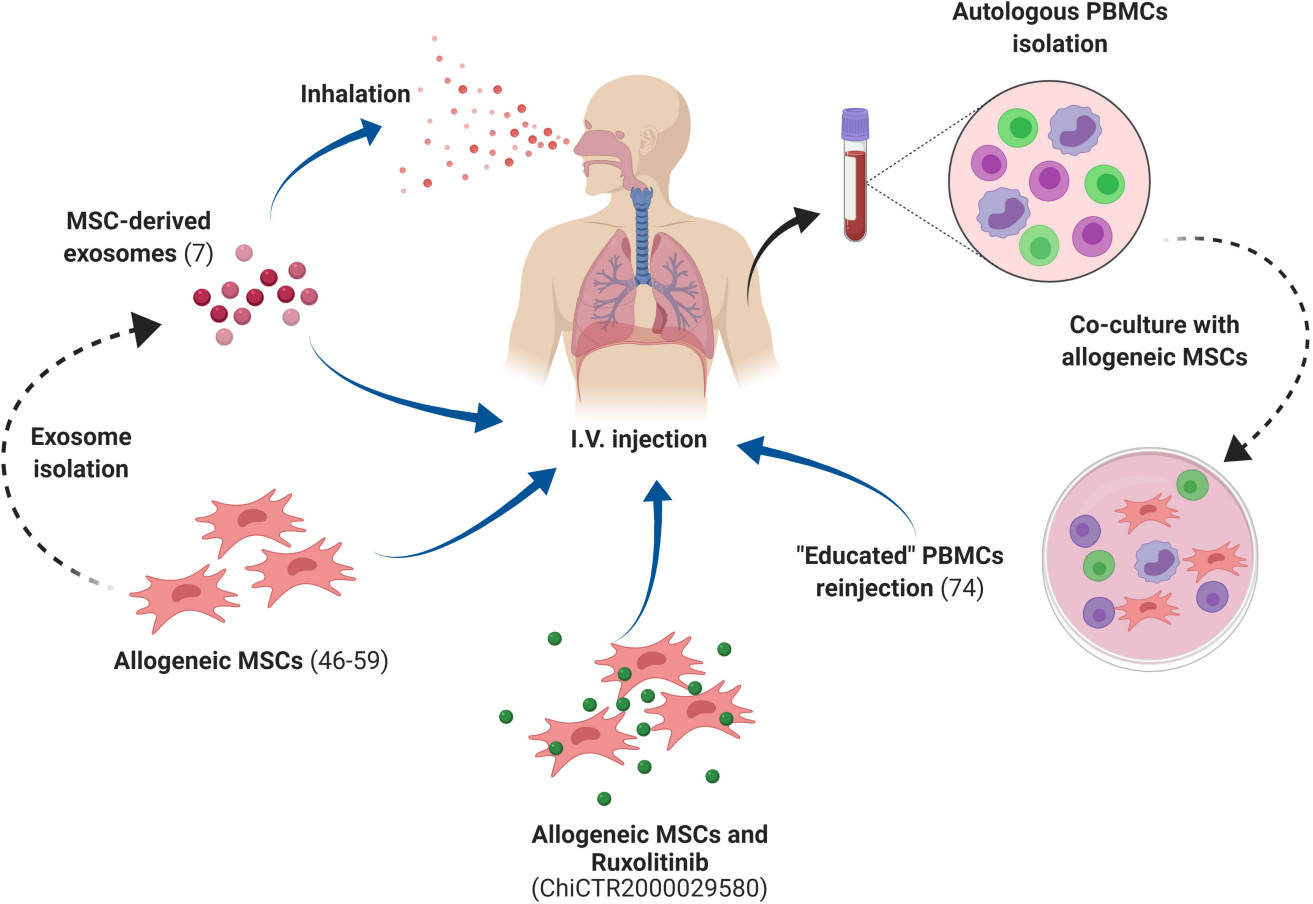
MSC-based strategies to treat COVID-19 under investigation in current clinical trials. Most clinical trials are based on the intravenous injection of allogeneic MSCs to treat COVID-19 patients. Other strategies include the isolation of MSC-derived exosomes, applied through inhalation, and also the temporary co-culture of autologous polymorphonuclear cells (PBMCs) collected from COVID-19 patients with allogeneic MSCs, followed by the injection of the so-called “educated” autologous PBMCs back to the COVID-19 patient. MSCs, Mesenchymal Stem Cells; PBMCs, Peripheral Blood Mononuclear Cells. Created with BioRender.com.

Using the terms “COVID-19” and “stem cells” we found studies in the ClinicalTrials.gov database (June, 2020). We complemented our search by assessing the complete list of COVID-19 clinical trials registered in the World Health Organization International Clinical Trials Registry Platform, and searching for studies which included the term “stem cells.” We finalized our search by accessing the European Union Clinical Trials Register (https://www.clinicaltrialsregister.eu). Studies that were withdrawn will not be mentioned in the present manuscript. Two observational studies were also removed from the list. We ended up with 69 clinical studies, 60 of which aim to investigate the therapeutic efficacy of MSCs to treat COVID-19. One trial aims to investigate the use of mononuclear cells, one trial investigates PRP in addition to stem cells, and six trials focus on the use of MSC-derived exosomes/conditioned media. One study aims to investigate the therapeutic benefits promoted by the combination of MSCs with ruxolitinib (Supplementary Table 1). One trial proposes the use of human Embryonic Stem Cells to produce “Immunity- and matrix-regulatory cells (NCT04331613). One trial focuses on using MSCs as “educators” of patients' mononuclear cells. By educated cells, researchers refer to mononuclear cells briefly co-cultured with MSCs *ex vivo*. As revised by Zhao (71), such a co-culture procedure leads to decreased expression of costimulatory molecules by T cells, increased Treg generation, and TGF-b1 synthesis. Educated cells have shown to be safe in clinical studies and to impose effective anti-inflammatory effects. The strategies under investigation based on Mesenchymal Stem Cells are illustrated in Figure 2, and involve different routes of administration, as well as the use of both MSCs, as well as their products, such as exosomes.

As expected, China hosts almost 50% of the detected trials (32 out of 69), but countries like the United States, Spain, France, Jordan, Turkey, Egypt, and Brazil are also executing clinical trials in the theme, underscoring the capacity of local scientists from different parts of the globe to respond to the present scenario. Furthermore, such data also reveals the capacity of each country to enable scientists and companies to rapidly initiate complex clinical studies, involving advanced therapy products. In Brazil, for instance, the regulatory framework is very recent (latest document being released by ANVISA in January 2020), even though the country has already important experience in the field of clinical application of stem cells (Brazilian institutions have already registered 98 clinical trials involving the use of stem cells, according to ClinicalTrials.gov. The first trial was registered in 2005).

Cell dose and proposed regimens varied among studies, but, importantly, we note that we could not find the number of injected cells in some studies. Considering the information we could gather from the aforementioned sources, the number of infused MSCs ranged between 0.5 × 10^6^ and 1 × 10^7^ cells/kg. Few studies proposed single doses of 3 × 10^7^, 4 × 10^7^, and 2 × 10^8^ cells, regardless of the weight of the patient. Most frequently, the mentioned doses will be injected three or even four times, during short time intervals of 2 or 3 days. All studies will perform intravenous cell infusion of cells. Two studies focusing on the use of MSC-derived exosomes have opted for inhalation routes, while the others propose intravenous infusion. Taken together, the studies are using relatively high numbers of cells in very short periods between doses, compared to most MSC clinical trials performed so far (72). High MSC doses are only assessed in a short time frame if derived from allogeneic MSC banks, which is the case in the cited studies regarding COVID-19.

The source of the MSCs used in the trials is also a point of variability in the detected studies. The most common source observed was the umbilical cord/wharton's jelly (proposed in 28 studies), followed by adipose tissue (9 out of 69), bone marrow (9 out of 69), dental pulp (3 out 69), embryonic stem cells (2 out of 69) and olfactory mucosa, placenta, and menstrual blood (1 study each). In 11 studies, the MSC source was unclear. Finally, 2 studies used cardiosphere stem cells, and the others did not involve stem cells directly (educated cells, conditioned media, etc).

Currently, it has not been established that any specific adult mesenchymal stem cell source is clearly superior to others (38). Nevertheless, a recent meta-analysis study by McIntyre et al. suggested that bone marrow- and cord blood-derived MSCs promoted slightly better therapeutic results than adipose tissue-derived MSCs in preclinical studies of acute lung injury (73). They also point to superior results following intratracheal administration of MSCs, compared to intravenous and intraperitoneal routes. Even though COVID-19 is mainly acknowledged as a respiratory syndrome, its systemic effects beyond the respiratory tract have also come to light (30, 74, 75). Such notion differs from the preclinical studies focused on lung injuries only, and justifies the choice of systemic routes of administration of MSCs in the COVID-19 studies.

The McIntyre et al. meta-analysis (73) also presented data which suggest that allogeneic MSCs led to better results than xenogeneic, syngeneic and autologous counterparts. Another interesting feature of the executed analysis is the suggestion that the use of frozen MSCs also led to superior results compared to fresh cells. It is important to emphasize that the meta-analysis executed by McIntyre et al. is based on relatively small number of studies, preventing major conclusions. Nevertheless, considering the logistics of obtaining high numbers of MSCs for therapy, evidence incentivizing the use of allogeneic, frozen cells, are of great value to support the design of clinical studies based on the use of allogeneic MSC cryobanks.

Most studies are investing in randomized trials (47 out of 69 studies), but 21 out of 69 trials have control groups that do not receive placebo (only local routine treatment), and 19 out of 69 studies lack control groups altogether. Only 29 out of 69 studies include placebo groups. Understandably, placebo-controlled clinical studies are controversial, especially when withholding treatment poses a significant risk for patients (76). Still, considering that most studies underway focus on the investigation of MSCs as add-on therapies, this means that most placebo groups would receive routine treatment, undermining ethical considerations.

Previous experience has taught valuable lessons regarding the importance of randomized studies, including control groups (77). Therefore, the percentage of the gold standard, randomized and double-blind clinical trials can be considered below expectations.

In the last 20 years of clinical experience with MSCs, it is beyond dispute that MSC therapy is safe (78, 79). As published by Thomson et al. (80), data derived from 55 clinical studies involving almost 3,000 patients who received MSC therapy indicate that the procedure is safe in the short-, medium- and long-term. Nevertheless, it is frequently difficult to correlate cellular therapies with long-term adverse effects.

Since the COVID-19 pandemic is recent, it does not come as a surprise that clinical evidence supporting the use of MSCs to treat the disease are still scarce. So far, few clinical studies have been published describing the results of MSC treatment for COVID-19 (Supplementary Table 2).

The study by Leng et al. (26) presented partial results of clinical trial ChiCTR2000029990, registered in the Chinese Clinical Trial Registry System (http://www.chictr.org.cn/index.aspx). The publication describes the 14-day outcome of 10 COVID-19 patients. Considering the 3 patients with severe COVID-19 who received placebo infusions, unfortunately, one died, one presented acute respiratory distress syndrome, and one presented severe COVID-19. Regarding the 7 patients who received MSC treatment, therapeutic benefits were mainly observed in the four patients with severe disease, and also in the single patient with critically severe disease, in contrast to the 2 MSC-treated COVID-19 patients who were not classified as severe. All MSC-treated patients survived and presented positive outcomes, 3 of them were discharged, and the authors mentioned no adverse effects.

More recently, in a company announcement, Mesoblast described no adverse effects and positive clinical data obtained in march-april 2020 regarding 12 ventilator-dependent COVID-19 patients who received two MSC infusions 4 days apart from each other (81). Among treated subjects, 10 (83%) survived and 9 (75%) came off ventilator during the period of observation. In the document, the company mentions two studies which describe contemporaneous data of 12% survival among ventilator-dependent COVID-19. Since the clinical study is still ongoing, it will be important to see the final results of the study, which includes a placebo control group and will, therefore, be able to provide more reliable control data.

Also during April 2020, the compassionate use of cardiosphere-derived cells (CDCs) was investigated by the teams of Cedars-Sinai Medical Center and Capricor Inc (82). According to the authors of the study, CDCs are much different from the controversial cardiac progenitor cells, and have already been investigated in more than 200 patients, presenting anti-inflammatory and immunomodulatory capacity. In the manuscript, the authors present the data regarding 6 ventilator-dependent COVID-19 patients, of which 100% (6 out of 6) survived, and 66.6% (4 out of 6) weaned from respiratory support and were discharged. Ferritin, lymphocyte counts tended toward normalization in most patients. A control group (*n* = 34) of critical COVID-19 patients with similar baseline characteristics, and simultaneously hospitalized at the same study site, was retrospectively analyzed and presented 18% mortality.

Two studies described data regarding individual COVID-19 patients who received umbilical cord-derived MSCs in different regimens. Zhang et al. performed the infusion of a single dose of 1 million cells/kg in a severe COVID-19 patient (83), who significantly benefited from the therapy and was discharged 7 days following the procedure. Liang et al. (84) treated a critically ill COVID-19 who failed to respond to previous glucocorticoid, antiviral (lopinavir/ritonavir, IFN-α inhalation and oseltamivir) and antibiotics (moxifloxacin, Xuebijing, methylprednisolone, and immunoglobulin), with three doses of 50 million MSCs. No adverse effects were observed, and the patient presented clinical and biomarker improvements after the second cellular infusion. Two days following the third cellular infusion, the patient left ICU.

In recognition of the value of the paracrine signaling on stem cell mechanism of action, stem cell derived exosomes were also investigated for COVID-19 management. In the study by Sengupta et al. (85), 24 COVID-19 patients received a single dose of ExoFlo. Patients were separated in cohorts according to clinical parameters. One patient presented dyspnea, 20 patients presented hypoxemia and required non-invasive oxygen support, and 3 patients presented respiratory failure and depended on mechanical ventilation. All patients received standard treatment of the institution at the time, composed of hydroxychloroquine and azhytrominin. Exosome-treated patients also received ExoFlo, which is a product of Direct Biologics, composed of exosomes derived from bone marrow MSCs and containing immunomodulatory molecules, growth factors and chemokines (https://directbiologics.com/growth-factors/). The amount of exosomes contained in the applied dose is unclear, but no adverse effects were registered related to the exosome treatment and clinical benefits were observed. Of the 24 patients which received ExoFlo, only 4 expired and 3 remained critically ill. Seventeen recovered, and therapeutic benefits peaked after 3–4 days from infusion, suggesting the possibility of performing more than one infusion.

As discussed by Leng et al. (26), it is important to point out that–different from lung alveolar and endothelial cells -, bone-marrow cells lack ACE-2 receptors. Therefore, it is possible that bone marrow derived-MSCs lack ACE-2 receptors, but Leng et al. were the first to show experimental evidence in this sense (i.e., RNASeq analysis), suggesting that the infused cells would not further replicate SARS-CoV-2. At this point, the expression of ACE-2 in stem cells of other sources is unclear.

As discussed, the rationale for using MSCs to treat COVID-19 is to revert the complex cytokine storm elicited by the SARS-CoV-2, similar to what has already been described for other viruses (86). In the scenario in which a single limited intervention may fail to revert the complex pathophysiology of COVID-19, the MSCs constitute a highly appealing therapeutic strategy. As aforementioned, MSCs cells act in several different processes of innate and adaptive immunity, possibly leading to changes in the overall activation of the immune system in patients with inflammatory diseases. MSCs also seem to exert therapeutic benefits by secreting anti-apoptotic and regenerative factors.

In the study of Leng et al. different from the placebo controls, severe and critically severe COVID-19 patients who received MSC infusion presented a significant increase in Tregs, and also a decrease in overactivated T and NK cells. Soluble factor analysis in the patients‘ serum revealed that treated patients presented increased IL-10, IP-10, VEGF levels, and also lower TNF-a production, compared to untreated patients. After running RNASeq analysis in the MSCs used for therapy, the authors found that such cells present high mRNA levels of important anti-inflammatory and trophic factors. The other published studies also pointed to improvements in biochemical parameters, as well as cytokines. Most importantly, publications point to the safety and efficacy of the procedures, which led to significant clinical improvements in fever, pneumonia, diarrhea, oxygenation, etc, ultimately leading to mechanical ventilation weaning, and even hospital discharge.

## Concluding Remarks

Taken together, the present data reveal that stem cells and stem cell-derived strategies are currently under investigation as therapeutic tools to manage COVID-19, and also that the limited observations published so far point to the safety and efficacy of such therapies in the short-term, at least in severe and critically severe patients. The replication of the positive outcomes by independent groups was an important milestone recently achieved in the field. The current data, albeit preliminary and descriptive in nature, are promising. Nevertheless, the comparison between different therapeutic regimens, including various doses, MSC sources, and injection routes in the short-term, as well as in long-term, will provide valuable information about how to potentialize therapeutic outcomes. In the safety assessment side, patients need to be followed for long periods after stem cell therapy, since the angiogenic and immunomodulatory effects of MSCs could influence other outcomes, such as cancer progression (87).

Drawing any major conclusions regarding the use of stem cells and stem cell-derived products at this point is impossible, due to the small evidence accumulated so far. Moreover, the lack of full detailed data of published studies may compromise future analysis as well. In this sense, the fact that a few studies cited here present incomplete data regarding patients, clinical outcomes, and other parameters is an important limitation of our study. Nevertheless, such a limitation has also been detected as an issue in previous works in the theme (66, 88, 89).

Cell therapies are complex and present high costs; therefore, it will be paramount to carefully analyze the effect of such a therapeutic strategy according to sensitive parameters, such as time in ICU, time to recovery and duration of hospitalization. Still, advanced therapies are hardly seen as a unique alternative to manage a pandemic, but rather as an alternative to possibly save patients in severe or critically-severe conditions. Cell-free strategies under investigation are more easily scaled-up, and may simplify the logistics and regulatory path for commercial registration.

Preliminary data regarding the safety and efficacy of MSC therapy for COVID-19 is positive and has been obtained by independent groups. A second generation of trials adhering to rigorously designed blind, randomized, placebo-controlled protocols must now be pursued, with the aid of experienced clinical and basic science investigators.

The stem cell sector suffers from much hype and, unfortunately, unproven stem cell therapies for COVID-19 (and other disorders) are being offered worldwide, as alerted by the International Society for Cell & Gene Therapy (90), and more thoroughly reviewed by Turner (91). In such a rapid-paced field, systematic studies and meta-analysis will aid the scientific community to separate hype from hope and offer an unbiased position to the society.

## Author Contributions

FS-A, EM, AS-C, and JC researched data and wrote the manuscript. All authors contributed to the article and approved the submitted version.

## Conflict of Interest

The authors declare that the research was conducted in the absence of any commercial or financial relationships that could be construed as a potential conflict of interest.
