# Supplementary material for: Mesenchymal Stem Cells: A New Piece in the Puzzle of COVID-19 Treatment
Source: Front Immunol. 2020 Jul 3;11:1563. doi: 10.3389/fimmu.2020.01563 (PMC7347794; doi:10.3389/fimmu.2020.01563)
Supplement: Supplementary file 1 [file Table_1.DOCX]

**Table 1.** Ongoing clinical trials using stem cells to treat COVID-19.

| **Clinical trial identifier and characteristics** | **Phase** | **Intervention** | **Cell dose, route and other details** | **Investigated outcomes** | **Target patient enrollment** | **Control group** | **Country** |
| --- | --- | --- | --- | --- | --- | --- | --- |
| ***ChiCTR2000029990**, Interventional, Randomized, Multicentre, Placebo controlled, Parallel assignment | 1/2 | MSCs | Not informed | **Primary**: Improved respiratory system function (blood oxygen saturation) recovery time  **Secondary**: Not informed | 120 (60 control group) | Placebo including vehicle only | China |
| ***NCT04371393 -** Randomized, parallel design, placebo controlled, Triple masking (Participant, Investigator, Outcomes Assessor). | 3 | BM-MSCs (Remestemcel-L) as add-on therapy. | Two doses of 2x10⁶ MSC/kg, Intravenous, days 0 and 4. | **Primary:** Number of all-cause mortality.  **Secondary:** Number of days alive off mechanical ventilatory support; Number of adverse events; Number of participants alive at day 7, 14, 60, 90; Number of participants with resolution and/or improvement of ARDS on days 7, 14, 21 and 30; Change from baseline of the severity of ARD on days 7, 14, 21 and 30; Length of stay; Clinical Improvement Scale on days 7,14,21 and 30; Change in serum hs-CRP concentration on days 7,14,21 and 30; Change in IL-6 and IL-8 inflammatory marker level on days 7,14,21 and 30; Change in TNF-alpha inflammatory marker level on days 7,14,21 and 30. | 300 (150 each group) | Placebo (Plasma-Lyte) plus standard of care. | United States |
| ***Not informed A -** Compassionate use, non-randomized | NA | CAP-1002 (Allogeneic Cardiosphere-Derived Cells) as add-on therapy | Maximum of two doses of 150 X10^6^ cells, intravenous, 6 days apart (± 1 day). All patients received anti-IL6 or anti-IL6 receptor agents | clinical status at 30.7 days of hospitalization; CRP; Ferritin; IL-6; lymphocyte counts | 6 | Contemporaneous untreated group | United States |
| ***Not informed B -** Non-randomized, prospective, open-label | NA | ExoFlo - BM-MSC-derived exosomes as add-on therapy | Single dose of ExoFlo, intravenous. | Clinical status, partial PaO2/FiO2; oxygen requirement; CRP, ferritin, and D-dimer; neutrophil count; CD3+, CD4+, and CD8+ T lymphocytes count. | 27 | No | United States |
| ***Not informed C -** Non-randomized, prospective, single group, open label | NA | WJ-MSCs | Single dose of 1X10^6^ cells/kg, intravenous | Symptoms of fever, weakness, shortness of breath, and low oxygen saturation; acute infusion-related or allergic reactions, CT score, CD3+ T cell, CD4+ T cell, and CD8+ T cell count, CRP, IL-6, TNFa, RT-qPCR for SARS-CoV-2 | 1 | No | China |
| ***Not informed D** | NA | UC-MSCs | Three doses of 5X10^7^ cells at days 0, 3 and 6 | Creatinine, Dbil, Ibil, Tbil, Albumin, ALT, AST, CRP, PCT, D-dimer, proBNP, white blood cell count, neutrophils, lymphocytes. | 1 | No | China |
| **NCT04338347 -** Randomized, Double-Blind, Placebo-Controlled Study | NA | CAP-1002 (Allogeneic Cardiosphere-Derived Cells) | Maximum of two doses of 150 X10^6^ cells, intravenous, 6 days apart (± 1 day) | Safety with vital signs (heart rate, blood pressure, respiratory rate, and oxygen saturation), physical examinations, electrocardiograms, clinical laboratory testing (CBC, CMP, BNP, CRP, ESR, cytokine assay, viral load, troponin I, myoglobin, ferritin, procalcitonin, ABGs, and lipid panel), chest x-rays, and adverse events; Proteomic assay testing; Transthoracic echocardiograms; Use of any concomitant medications. | NA | Placebo | United States |
| **NCT04313322** - Interventional, Prospective, single group, open-label. | 1 | WJ-MSCs | Three doses of 1X10^6^/kg, intravenous, 3 days apart from each other. | **Primary:** Improvement of clinical symptoms including duration of fever, respiratory distress, pneumonia, cough, sneezing, diarrhea; Side effects; viral load; time to negative viral load.  **Secondary:** viral load;  time to negative viral load. | 5 | No | Jordan |
| **NCT04315987** - Interventional, Prospective, Non-randomized, single group, open-label | 1/2 | NestCell® as add-on therapy | Three doses of 1X10^6^/kg, intravenous, days 1, 3 and 7. | **Primary:** Disappear time of ground-glass shadow in the lungs.  **Secondary:** Rate of mortality within 28-days; Change of Clinical symptoms including duration of fever and respiratory;  Time of nucleic acid turning negative; CD4+ and CD8+ T cell count; Changes of blood oxygen; Side effects in the treatment group. | 66 | No | Brazil |
| **NCT04288102** - Interventional, Prospective, Randomized, Placebo controlled, Multi-center Phase 2 Clinical Trial, quadruple masking (Participant, Care Provider, Investigator, Outcomes Assessor). | 2 | MSCs as add-on therapy | Single dose of 4X10^7^ cells (≥70Kg) or 3X10^7^ cells (<70kg) , intravenous, days 0, 3, 6. | **Primary:** Size of lesion area and severity of pulmonary fibrosis by chest CT.  **Secondary:** Proportion of patients in each classification of clinical critical treatment index; Oxygenation index; Duration of oxygen therapy and hospitalization; Blood oxygen saturation; CD4+ T cell count and cytokine level; Side effects. | 90 (60 treatment and 30 placebo) | Placebo including vehicle only | China |
| **NCT04302519** - Interventional, Prospective, Non-randomized, single group, open-label | 1 | Dental Pulp MSCs as add-on therapy | Three doses of 1.0x10^6^ cells /kg, intravenously, days 1, 3, 7. | **Primary**: Disappear time of ground-glass shadow in the lungs.  **Secondary**: Absorption of Lung shadow absorption by CT Scan-Chest; Changes of blood oxygen. | 24 | No | Shanghai |
| **NCT04252118** - Interventional, Prospective, Non-Randomized, Parallel assignment, open-label. | 1 | MSCs as add-on therapy | Three doses of  3.0x10^7^ MSCs intravenous, days 0, 3, 6. | **Primary**: Size of lesion area by chest radiograph or CT; Side effects.  **Secondary**: Improvement of Clinical symptoms (fever and respiratory); Time of nucleic acid turning negative; rate of mortality within 28-days, CD4+ and CD8+ T cell count; alanine aminotransferase; C-reactive protein; Creatine kinase. | 20 (10 patients in each arm) | Routine treatment | China |
| **NCT04273646** - Interventional, Prospective, Randomized, Placebo controlled, parallel assignment, open-label | N.A. | UC-MSCs as add-on therapy | Four doses of  5.0X10^6^ cells/kg, intravenous, days 1, 3, 5, 7 | **Primary**: Pneumonia severity and oxygenation index.  **Secondary**: side effects in the UC-MSCs treatment group; 28-days survival; sequential organ failure assessment; C-reactive protein; Procalcitonin, lymphocyte count, CD3+; CD4+ and CD8+ T cell count; CD4+/CD8+ratio. | 48 (24 patients in each arm) | Placebo including vehicle only | China |
| **NCT04299152**, Interventional, Prospective, Randomized, two-arm, single-center, single masking (care provider). | 2 | Stem Cell Educated autologous immune cells | N.A., intravenous | **Primary**: Determine the number of Covid-19 patients who were unable to complete SCE Therapy.  **Secondary**: Percentage of activated T cells; Th17 cells; Chest imaging changes by computed tomography (CT) scan of the chest; viral load. | 20 | Routine treatment | China |
| **NCT04331613** - Interventional, Prospective, Single group assessment, open-label. | 1/2 | Immunity- and matrix-regulatory cells differentiated from clinical-grade human embryonic stem cells (hESCs) – CAStem. | Single dose of 3X10^6^, 5X10^6^ or 10X10^6^ cells/kg , intravenous. | **Primary**: Adverse reaction (AE) and severe adverse reaction (SAE); changes in lung image examination.  **Secondary**: Time to SARS-CoV-2 RT-PCR negative; Duration of fever; Changes of blood oxygen; Rate of all-cause mortality within 28 days; Lymphocyte count; Alanine aminotransferase; Creatinine; Creatine kinase; C-reactive protein; Procalcitonin; Lactate; IL-1beta; IL-2; IL-6; IL-8. | 9 | No | China |
| **NCT04269525** - Interventional, Prospective Non-randomized, single group assessment, open label. | 2 | UC-MSCs | Four doses of 9.9X10^7^cells, intravenous, days 1, 3, 5, 7. | **Primary**: oxygenation index.  **Secondary**: 28 day mortality; Hospital stay; viral load; Improvement of lung imaging examinations; White blood cell count; Lymphocyte count; Lymphocyte percentage; Procalcitonin; IL-2; IL-4; IL-6; IL-8; IL-10; TNF-α; γ-interferon. | 10 | No | China |
| **NCT04333368** - Interventional, Randomized, Placebo controlled, Parallel assignment, tryple masking (Participant, Care Provider, Investigator). | 1/2 | WJ-MSCs | Three doses of 1.0X10^6^ cells /kg, intravenously, days 1, 3, 5 | **Primary**: Respiratory efficacy evaluated by the increase in PaO2/FiO2 ratio from baseline to day 7 in the experimental group compared with the placebo group.  **Secondary**: Lung injury score; Oxygenation index; In-hospital mortality; Mortality; Ventilator-free days; Number of days between randomization and the first day the patient meets weaning criteria; Cumulative use and duration of use of sedatives and neuromuscular blocking agents ; ICU-acquired weakness and delirium; treatment-induced toxicity rate and adverse events; Quality of life at one year; Measurements of plasmatic cytokines (IL1, IL6, IL8, TNF-alpha, IL10, TGF-beta, sRAGE, Ang2); Anti-HLA antibodies. | 60 (20 treated, 40 placebo) | Placebo including vehicle only. | France |
| **NCT04276987** - Interventional, Prospective, Single group assignment, open-label. | 1 | Exosomes derived from allogeneic AT-MSCs (MSCs-Exo). | Five doses of 2.0X10^8^ nano vesicles, Days 1, 2, 3, 4, 5,  Aerosol inhalation. | **Primary**: AE and SAE; Time to clinical improvement.  **Secondary**: Number of patients weaning from mechanical ventilation; Duration (days) of ICU monitoring, vasoactive agents usage, mechanical ventilation supply; Number of patients with improved organ failure, improved organ failure; Rate of mortality. | 30 | No | China |
| **NCT04336254 –** Interventional, Single-center, Prospective, Randomised, Placebo controlled | 1/2 | Biological: allogeneic human dental pulp stem cells. | One dose of 3.0X10⁷ cells/dose intravenous, day 1, day 4 and day 7. | **Primary:** Time to Clinical Improvement.  **Secondary:** Lung Lesion by CT; Immune function of Th1 cytokines: IL-1β, IL- 2, TNF-a, ITN-γ; Th2 cytokines: IL- 4, IL- 6, IL- 10; Immunoglobulins: IgA, IgG, IgM, and total IgE; Lymphocyte counts: CD3+, CD4+, CD8+, CD16+, CD19+, CD56+; Time of SARS-CoV-2 test turns negative; Blood cell count and classification; Pulse oximetry; Respiratory rate; Body temperature; Number of the included patients with hDPSCs-related adverse events, e.g. liver or kidney function failure; C-reactive protein levels. | 20 | Placebo including vehicle only. | China. |
| **NCT04348435 -** Randomized, Placebo-Controlled, Double-Blinded. | 2 | AT-MSCs | Five doses of 2X10⁸ , 1X10⁸ or 5X10⁷ cells, intravenous, weeks 0, 2, 6, 10, and 14. | **Primary:** Incidence of hospitalization for COVID-19; Incidence of symptoms associated with COVID-19.  **Secondary:** Absence of upper/lower respiratory infection; Leukocyte differential; C Reactive protein; TNF alpha; IL-6; IL-10; Glucose; Calcium; Albumin; Total protein; Sodium; Total carbon dioxide ; Potassium; Chloride; BUN; Creatinine; Alkaline phosphatase; Alanine aminotransferase; Total bilirubin; White blood cells; Red blood cells; hemoglobin; hematocrit; mean corpuscular volume; mean corpuscular hemoglobin; mean corpuscular hemoglobin concentration; red cell distribution width; neutrophils; Lymphs; Monocytes; Eosinophils; Basophils; Absolute neutrophils; Absolute lymphs; Absolute monocytes; Absolute eosinophils; Absolute basophils; Immature granulocytes; Platelets; Prothrombin time; INR; SF-36; PHQ-9. | 100 | Placebo including vehicle only. | United States. |
| **NCT04366323 -** Multicenter, Randomized and Controlled, Open label | 1/2 | AT-MSCs | Two doses of 8X10⁷ cell/dose. | **Primary:** Safety of the administration of allogeneic mesenchymal stem cells derived from adipose tissue assessed by Adverse Event Rate; Efficacy of the administration of allogeneic mesenchymal stem cells derived from adipose tissue assessed by Survival Rate. | 26 | No intervention | Spain |
| **NCT04349631 -** Open Label, Single-Center, | 2 | AT-MSCs | Five infusions of cells, intravenous. | **Primary:** Incidence of hospitalization for COVID-19; Incidence of symptoms for COVID-19.  **Secondary:** Neutrophils; Absolute lymphs; Absolute monocytes; Absolute eosinophils; Absolute basophils; Immature granulocytes; Absolute Immature granulocytes; Platelets; Prothrombin time; INR; TNF alpha; Absence of upper/lower respiratory infection; Absence of upper/lower respiratory infection by hospitalization criteria; Glucose; Calcium; Albumin; Total Protein; Sodium; Total carbon dioxide; Potassium; Chloride; BUN; Creatinine; Alkaline phosphatase; Alanine aminotransferase; Aspartate aminotransferase; Total bilirubin; White blood cells; Red blood cells; Hemoglobin; Hematocrit; Mean corpuscular volume; Mean corpuscular hemoglobin; Mean corpuscular hemoglobin concentration; Red cell distribution width; Neutrophils; Lymphs; Monocytes; Eos; Basophils; Absolute in-6; Interleukin-10; C-reactive protein; SF-36; PHQ-9. | 56 | No | United States |
| **NCT04346368 -** Randomized, Placebo Controlled Trial, Parallel assignment, single masking (participant). | 1/2 | BM-MSCs | One dose 1X10⁶/kg, intravenously. | **Primary:** Changes of oxygenation index (PaO2/FiO2); Evaluation of pneumonia improvement; Side effects in the BM-MSCs treatment group.  **Secondary:** Clinical outcome; Hospital stay; CT Scan; Changes in viral load; Changes of CD4+, CD8+ cells count and concentration of cytokines; Rate of mortality within 28-days; Changes of C-reactive protein. | 20 | Placebo | China |
| **NCT04382547 -** Non-randomized, Parallel assignment, open label | 1/2 | Olfactory Mucosa MSCs (OM-MSCs) | Number and dose of cells were not informed, intravenous. | **Primary:** Number of cured patients; Number of patients cured, assessed by PCR in addition to chest CT scan.  **Secondary:** Number of patients with treatment-related adverse events. | 40 | Routine treatment | Belarus |
| **NCT04366063 -** Randomized, Parallel assignment, Open label | 1 | MSCs and EV-MSCs as add-on therapy. | **Cell therapy protocol 1**: Two doses of MSCs 100X10⁶ (±10%) at Day 0 and Day 2.  **Cell therapy protocol 2:** Two doses of MSCs 100×10⁶ (±10%) at Day 0 and Day 2, intravenously plus two doses of EVs at Day 4 and Day 6. | **Primary:** Adverse events assessment; Number of participants with treatment-related adverse events as assessed by CTCAE v4.0; Blood oxygen saturation; Evaluation of Pneumonia Improvement.  **Secondary:** Intensive care unit-free days; Clinical symptoms; Improvement of clinical symptoms including duration of fever, respiratory distress, pneumonia, cough, sneezing; Respiratory efficacy; Biomarkers concentrations in plasma. | 60 (20 each group) | Routine treatment | Iran |
| **NCT04339660 -** Randomized, Placebo controlled, Parallel assignment, triple masking (Participant, Care Provider, Outcomes Assessor). | 1 | UC-MSCs | One dose of 1X10⁶/kg. | **Primary:** The immune function (TNF-α 、IL-1β、IL-6、TGF-β、IL-8、PCT、CRP); Improvement and recovery time of inflammatory and immune factors; Blood oxygen saturation.  **Secondary:** Rate of mortality within 28-days; Size of lesion area by chest imaging; CD4+ and CD8+ T cells count; Peripheral blood count recovery time; Duration of respiratory symptoms (fever, dry cough, difficulty breathing, etc.); Indirect response to lung function; COVID-19 nucleic acid negative time. | 30 (15 each group) | Placebo including vehicle only. | China |
| **NCT04392778 -** Randomized, parallel assignment, Placebo controlled, quadruple masking (Participant, Care Provider, Investigator, Outcomes Assessor) | 1 | UC-MSCs | One dose of 3X10⁶ cells/kg intravenous on day 0, 3 and 6. | **Primary:** Clinical improvement; Improvement of clinical symptoms related to Covid-19 infection (fever, pneumonia, shortness of breath).  **Secondary:** Lung damage improvement; Improvement of lungs assessed by CT Scan; Sars-Cov-2 viral infection laboratory test; Negative, measured by RT-PCR laboratory tests for the virus; Blood test. | 30 (10 each group) | Placebo: Intravenous saline injection. | Turkey |
| **NCT04371601 -** Randomized, Parallel assignment, Open lable | 1 | UC-MSCs as add-on therapy | Four doses of 10⁶/Kg, once every 4 days. | **Primary:** Changes of oxygenation index (PaO2/FiO2), blood gas test; Improvement of pulmonary function.  **Secondary :** Detection of TNF-α levels, IL-10 levels; Detection of immune cells that secret cytokines, including CXCR3+, CD4+, CD8+, NK+ cells, and regulatory T cells (CD4 + CD25 + FOXP3 + Treg cells); Changes of oxygenation index (PaO2/FiO2) ,blood gas test; Changes of c-reactive protein and calcitonin. | 60 | Routine therapy. | China |
| **NCT04355728 -** Randomized, Parallel assignment, single masking Single (Outcomes Assessor). | 1/2 | UC-MSCs as add-on therapy | Two doses of 100x10⁶cells/infusion, intravenous. | **Primary:** Incidence of pre-specified infusion associated adverse events; Incidence of Severe Adverse Events.  **Secondary:** Survival rate after 90 days post first infusion; Ventilator-Free Days; Change in Oxygenation Index (OI); Plat-PEEP; Sequential Organ Failure Assessment (SOFA) Scores; Small Identification Test (SIT) scores; As assessed via serum blood samples; C-Reactive Protein levels; As assessed via serum blood samples; Arachidonic Acid (AA)/Eicosapentaenoic Acid (EPA) Ratio; As assessed via serum blood samples; D-dimer levels; As assessed via serum blood samples; 25-Hydroxy Vitamin D levels; As assessed via serum blood samples; Alloantibodies levels; As assessed via serum blood samples; Blood white cell count; As assessed via serum blood samples; Platelets count. | 24 (12 each group) | Routine treatment | United States |
| **NCT04362189 -** Randomized, Placebo-Controlled, Single Center, Quadruple masking (Participant, Care Provider, Investigator, Outcomes Assessor) | 2 | AT-MSCs | Four intravenous infusions at 100 X 10⁶ cells at days 0, 3, 7, and 10. | **Primary:** D-dimer; Interleukin-6; C Reactive protein; Oxygenation; PCR test SARS-CoV-2.  **Secondary:** EKG qt interval; Leukocyte differential; TNF alpha; Glucose; Calcium; Albumin; Total protein; Sodium; Total carbon dioxide; Potassium; Chloride; BUN; Creatinine; Alkaline phosphatase; Alanine aminotransferase; Total bilirubin; White blood cells; Red blood cells; Hemoglobin; Hematocrit; Mean corpuscular volume; Mean corpuscular hemoglobin; Mean corpuscular hemoglobin concentration; Red cell distribution width; Neutrophils; Lymphs; Monocytes; Eosinophils; Basophils; Absolute neutrophils; Absolute lymphs; Absolute monocytes; Absolute eosinophils; Absolute basophils; Immature granulocytes; Platelets; Prothrombin time; INR; NK cell surface antigen (CD3-CD54+); CD4+/CD8+ ratio; IL-10; VEGF; Myoglobin; Troponin; Creatinine kinase; Serum ferritin; Adverse events; Point ordinal scale. | 100 (50 each group) | Placebo: Intravenous saline injection. | United State |
| **NCT04390152 -** Randomized, Parallel Assignment, Quadruple (Participant, Care Provider, Investigator, Outcomes Assessor) | 1/2 | WJ-MSCs as add-on therapy | Two doses of 50X10⁶, intravenous. | **Primary:** Intergroup mortality difference with treatment.  **Secondary:** Number of patients with treatment related adverse events; Difference in days of mechanical ventilation between groups; Median reduction of days of hospitalization; Median reduction of days of oxygen needs; Difference between "Sequential Organ Failure Assessment" score between groups; Difference between median Murray score between groups; Difference in APACHE II score between groups; The effect of WJ-MSC in the APACHE II score will compared between the two groups; Difference in lymphocyte count between groups; Evaluation of the effect of WJ-MSC in lymphocyte count measured in absolute number/mm3; These laboratory measures have been associated with COVID 19 severity; Changes in C reactive protein concentration between groups; Evaluation of the effect of WJ-MSC in C reactive protein concentration between the two groups, measured in mg/dl; Highest levels have been associated with COVID 19 severity and inflammation; Changes in D dimer concentration; Changes in ferritin concentration; Evaluation of the effect of WJ-MSC in ferritin compared between the two groups, measured in nanograms/ml; These laboratory measures have been associated with COVID 19 infection and severity; Changes in lactate dehydrogenase concentration; Evaluation of the effect of WJ-MSC in LDH compared between the two groups, measured in units/liter; Impact on interleukin 6, 8, 10 concentrations between groups; Impact on tumor necrosis factor alpha concentrations between groups. | 40 | Routine treatment | Colombia |
| **NCT04293692 -** Randomized, Placebo controlled, Parallel Assignment, Triple (Participant, Care Provider, Outcomes Assessor). | Not Applicable | UC-MSCs | Four doses of 0.5X10⁶ /kg, intravenously at Day1, Day3, Day5, Day7. | **Primary:** Size of lesion area by chest imaging; Blood oxygen saturation.  **Secondary:** Rate of mortality within 28-days; Sequential organ failure assessment; Side effects in the UC-MSCs treatment group; Electrocardiogram, the changes of ST-T interval mostly; Concentration of C-reactive protein C-reactive protein, immunoglobulin; CD4+ and CD8+ T cells count; Concentration of the blood cytokine (IL-1β, IL-6, IL-8,IL-10,TNF-α); Concentration of the myocardial enzymes. | 0 | Placebo including vehicle only. | China. |
| **NCT04348461** - Randomized, Parallel assignment, Controlled, Multicenter, Quadruple masking (Participant, Care Provider, Investigator, Outcomes Assessor). | 2 | AT-MSCs | Two doses of 1.5 X 10⁶/kg | **Primary:** Efficacy of the administration of allogeneic mesenchymal stem cells derived from adipose tissue assessed by Survival; Safety of the administration of allogeneic mesenchymal stem cells derived from adipose tissue assessed by Adverse Event. | 100 (50 each group) | Routine treatment. | Spain |
| **NCT04377334 -** Randomized, Parallel assignment, Open label. | 2 | BM-MSCs | Not informed | **Primary:** Lung injury score.  **Secondary:** D-dimers; phenotype; pro-resolving lipid mediators; cytokines; chemokines; Survival; extubation; lymphocyte subpopulations; SARS-CoV-2-specific antibody titers; complement molecules. | 40 (20 each group). | No Intervention. | Germany |
| **NCT04390139 -** Randomized, Parallel, Placebo-controlled, Multicentre, Quadruple masking (Participant, Care Provider, Investigator, Outcomes Assessor). | 1/2 | WJ-MSCs as add-on therapy. | Two doses, 1X10⁶/kg, intravenous. | **Primary:** All-cause mortality at day 28.  **Secondary:** Safety of WJ-MSC; Need for treatment with rescue medication; Need and duration of mechanical ventilation; Ventilator free days; Evolution of PaO2 / FiO2 ratio; Evolution of the SOFA index; Evolution of the APACHE II score; Duration of hospitalization; Evolution of markers of immune response (leucocyte count, neutrophils); Feasibility of WJ-MSC administration; Feasibility of WJ-MSC administration; Evolution of disease biomarker: polymerase chain reaction (RT-PCR); Evolution of disease biomarker: lactate dehydrogenase (LDH); Evolution of disease biomarker: D-dimer; Evolution of disease biomarker: Ferritin. | 30 | Placebo | Spain |
| NCT04341610 - Randomized, Parallel, Placebo-controlled, Quadruple (Participant, Care Provider, Investigator, Outcomes Assessor). | 1/2 | AT-MSCs | Single 100X10⁶ cells, route of administration not informed. | **Primary:** Changes in clinical critical treatment index.  **Secondary:** Days of respirator treatment; Improvement of clinical symptoms including duration of fever and respiratory need; Mortality; Marker of Immunological function -CD4+ and CD8+ T cell count; C-reactive protein and leucocyte; Cytokine profile; Glomerular Filtration Rate; Duration of hospitalization. | 0 | Placebo including vehicle only. | Denmark |
| **NCT04400032 -** Non-randomized, sequential assignment, open label. | 1 | BM-MSCs | Three doses of 25 X10⁶ cells, 50 X10⁶ cells, or 90 X10⁶ cells, intravenous. | **Primary:** Number of Participants With Treatment-Related Adverse Events as Assessed by CTCAE v4.0.  **Secondary:** Number of Participants alive by Day 28; Number of Participants with ventilator-free Days by Day 28. | 9 | No | Canada |
| **NCT04398303 -** Randomized, Placebo-Controlled, Double masking (Participant, Investigator) | 1/2 | WJ-MSCs and WJ-MSC-conditioned media. | Single dose of 1X10⁶ cells / kg or 100 ml of conditioned media, intravenous. | **Primary:** Mortality at day 30  **Secondary:** Ventilated Subjects - Ventilator Free Days; Ventilated Subjects - Improvement in Ventilator Settings; High-Flow O2 Support Subjects - Step-Down O2 Therapy; High Flow O2 Support Subjects - Respiration Rate; Both Ventilated and High-Flow O2 Support Subjects - ICU-Free Days; Both Ventilated and High-Flow O2 Support Subjects - Pulmonary Function Improvement; Both Ventilated and High-Flow O2 Support Subjects - Increased Berlin Score. | 70 | Placebo including vehicle only (MEM-α). | United States |
| **NCT04393415 -** Randomized, parallel, Double masking (Participant, Outcomes Assessor). | Not Applicable | Cord blood stem cells or PRP. | Not informed | **Primary:** The number of patients with positive covid 19 who will improve after receiving stem cells. | 100 | No intervention and PRP. | Egypt |
| **NCT04397796 -** Randomized, Parallel, Placebo-Controlled, Quadruple masking (Participant, Care Provider, Investigator, Outcomes Assessor). | 1 | BM-MSCs | Not informed | **Primary:** Incidence of AEs; Mortality; Death; Number of ventilator-free days.  **Secondary:** Improvement of one category; Sequential Organ Failure Assessment (SOFA); Oxygen; Hospitalization; Incidence of SAEs. | 45 | Placebo including vehicle only. | United States |
| **NCT03042143 -** Open Label Dose Escalation Phase 1 Trial Followed by a Randomized, Double-blind, Placebo-controlled Phase 2 . | 1/2 | WJ-MSCs (CD362 enriched). | Single dose of up to 400X10⁶ cells. | **Primary:** Oxygenation index (OI); Incidence of Serious Adverse Events (SAEs).  **Secondary:** Oxygenation index; Sequential Organ Failure Assessment (SOFA) score; Respiratory compliance (Crs); Partial pressure of arterial oxygen to the fraction of inspired oxygen ratio (P/F ratio); Driving Pressure; Extubation and reintubation; Ventilation free days at day 28; Length of ICU and hospital stay; 28-day and 90-day mortality**.** | 75 | Placebo including vehicle only. | United Kingdom |
| **NCT04345601-** Single group, open label | 1 | BM-MSCs | Single dose of 1X10⁸ MSCs, intravenous. | **Primary:** Incidence of unexpected adverse events; Improved oxygen saturations ≥93%.  **Secondary:** Decrease in oxygen supplementation by non-invasive or invasive interventions; Frequency of progression to mechanical ventilation or ECMO; Duration of mechanical ventilation; Duration of ICU stay; Duration of hospital stay; All-cause mortality at day 28. | 30 | No | United States |
| **NCT04361942-** Randomized, Parallel, Placebo-controlled, Triple masking (Participant, Care Provider, Investigator). | 2 | MSCs | Single dose, 1X10^6^ cells/Kg, Intravenous. | **Primary:** Proportion of patients who have achieved withdrawal of invasive mechanical ventilation; rate of mortality.  **Secondary:** Proportion of patients who have achieved clinical response; Proportion of patients who have achieved radiological responses. | 24 | Placebo including vehicle only. | Spain |
| **NCT04333368 -** Randomized, Parallel, Placebo-controlled, Triple masking (Participant, Care Provider, Investigator). | 1/2 | WJ-MSCs | Three doses of 1X10^6^ cells/Kg, Intravenous, at days 1, 3 and 5. | **Primary:** Respiratory efficacy evaluated by the increase in PaO2/FiO2 ratio from baseline to day 7 in the experimental group compared with the placebo group.  **Secondary:** Lung injury score; Oxygenation index; In-hospital mortality; Mortality; Ventilator-free days; Number of days between randomization and the first day the patient meets weaning criteria o Number of days between randomization and the first day the patient meets PaO2/FiO2 > 200 (out of a prone positioning session); Cumulative use of sedatives; Cumulative duration of use of sedatives; Cumulative duration of use of neuromuscular blocking agents (other than used for intubation); Cumulative use of neuromuscular blocking agents (other than used for intubation); Treatment-induced toxicity rate and adverse events up to day 28; Quality of life at one year (EQ5D-3L quality of life questionnaire); Measurements of plasmatic cytokines (IL1, IL6, IL8, TNF-alpha, IL10, TGF-beta, sRAGE, Ang2) level; Anti-HLA antibodies plasmatic dosage. | 40 (20 each group) | Placebo including vehicle only. | France |
| **NCT04389450 -** Randomized, Placebo-Controlled, Multicenter, Parallel, Quadruple (Participant, Care Provider, Investigator, Outcomes Assessor). | 2 | PLX-PAD (placental mesenchymal-like adherent stromal cells) | Interval high dose: Two cellular infusions  Interval low dose: one cellular infusion and one placebo infusion.  Control A: two infusions of placebo. All intramuscular, days 1 and 8. High dose: single cellular infusion.  Control B: single placebo infusion All intramuscular. | **Primary:** Number of ventilator free days.  **Secondary:** All-cause mortality; Duration of mechanical ventilation. | 140 | Placebo solution for injection. | United States |
| **NCT04367077-** Randomized, Placebo-Controlled, Multicenter, Sequential assignment, Quadruple (Participant, Care Provider, Investigator, Outcomes Assessor). | 2/3 | BM-MSCs (MultiStem). | Dose was not informed, Intravenous. | **Primary:** Ventilator-Free Days; Safety and Tolerability as measured by the incidence of treatment-emergent adverse events as assessed by CTCAE v5.0.  **Secondary:** All-cause mortality; Ranked hierarchical composite outcome of alive and ventilator-free; Ventilator-free day. | 400 | Placebo including vehicle only. | United States |
| **ChiCTR2000031319**, Interventional, Prospective, Randomized, Placebo controlled, Parallel assignment.  Masking: not informed. | N.A. | Dental Pulp MSCs as add-on therapy. | Not informed. | **Primary**: Time to clinical improvement.  **Secondary**: Immune biomarkers, Degree of Lung Lesion by CT; Time of SARS-CoV-2 test turns negative; Blood test; SPO2; RR; Body temperature; Biochemical tests. | 20 (10 placebo) | Routine treatment plus placebo | China |
| **ChiCTR2000030866**, Observational, Prospective, Single arm,  open-label. | 0 | UC-MSCs | Three doses of 1X10^6^ cells/kg, intravenous, days 0, 3, 6 . | **Primary**: Oxygenation index; Conversion rate from serious to critical patients; Conversion rate and conversion time from critical to serious patients; Mortality in serious and critical patients.  **Secondary**: Low-dose spiral CT of the lung; Peak Flow meter; viral load; Peripheral blood lymphocyte subsets; Cytokine detection; Sequential organ failure assessment; APACHE II score; Acute lung injury score; Blood routine; C-reactive protein; liver and kidney function; myocardial enzymes; coagulation function; D-dimer; arterial blood gas analysis; Respiratory function support types and parameters; tumor markers. | 30 | No | China |
| **ChiCTR2000030835**, Prospective, Interventional, Multicentre, Single arm, Non-randomized. | New Treatment Measure Clinical Study. | UC-MSCs | Three doses of 1X10^6^ cells/kg, intravenous, every 2 days or three doses of  2X10^6^ cells/kg, intravenous, every 3 days. Fourth application is optional. | **Primary**: Not informed  **Secondary**: C-reactive protein; Detection of lymphocyte subsets; urinalysis; Procalcitonin; Routine blood test; Chest CT; cytokine; Blood biochemistry | 20 (10 low dose and 10 high dose regimen) | No | China |
| **ChiCTR2000030300**, Interventional, single center, Non-randomized | 1 | UC-MSCs | Not informed | **Primary**: Time to disease recovery; Exacerbation (transfer to RICU) time  **Secondary**: not informed. | 9 | No | China |
| **ChiCTR2000030261**, Interventional, Parallel assignment, Randomized. | 0 | MSC exosomes | Aerosol inhalation of exosomes | **Primary**: Lung CT  **Secondary**: Nucleic acid detection of pharyngeal test; Leukocytes and lymphocytes in blood routine | 26 (13 control group) | Routine treatment | China |
| **ChiCTR2000030173**, Interventional, Randomized, Parallel assignment. | 0 | UC-MSCs | Not informed | **Primary**: pulmonary function; Novel coronavirus pneumonic nucleic acid test  **Secondary**: pulmonary CT; chest radiography | 60 (30 control group) | Routine treatment | China |
| **ChiCTR2000030138**, Interventional, Randomized, Placebo controlled, Parallel assignment, double-blind. | 2 | UC-MSCs | Intravenous | **Primary**: Clinical index | 60 (30 control group) | Routine treatment and placebo | China |
| **ChiCTR2000030116**, Interventional, Randomized. | Not informed | MSCs | Two different doses of UC-MSCs | **Primary**: Time to leave ventilator on day 28 after receiving MSCs infusion  **Secondary**: Not informed | 16 (two MSC doses) | No/not informed | China |
| **ChiCTR2000030088**, Prospective, Parallel assignment, Placebo controlled, Randomized. | 0 | WJ-MSCs | 1X10^6^ cells/kg, intravenous | **Primary**: The nucleic acid of the novel coronavirus is negative; CT scan of ground glass shadow disappeared  **Secondary**: Not informed | 40 (20 control group) | Placebo including vehicle only | China |
| **ChiCTR2000030020**, Interventional, Non-randomized (sequential), single-centre, single-arm. | Not informed | MSCs | Not informed | **Primary**: Coronavirus nucleic acid markers negative rate; Symptoms improved after 4 treatments; Inflammation (CT of the chest).  **Secondary**: Trough and peak of pulmonary function FEV1; Lymphocyte subpopulation changes. | 20 | No | China |
| **ChiCTR2000029606**, Randomized, open-label | 0 | Human Menstrual Blood-Derived Stem Cells and artificial liver in the treatment of Acute lung Injury. | Conventional treatment followed by Intravenous infusion of Human Menstrual Blood-derived Stem Cells preparations (A) or Conventional treatment (B) or Artificial liver therapy+conventional treatment (C) or Artificial liver therapy followed by Intravenous infusion of Human Menstrual Blood-derived Stem Cells preparations+conventional treatment (D). | **Primary**: Mortality  **Secondary**: Improvement rate in patients; Incidence of shock; Incidence of multiple organ failure; Days in hospital, days in ICU; Non-invasive ventilation modes and parameters; Intubation-assisted ventilation modes and parameters; Extracorporeal membrane oxygenation patterns and parameters. | 63 (18 group A, 25 group B, 10 group C, 10 group D). | Routine treatment. | China |
| **ChiCTR2000029580** - Prospective, single blind, Randomized controlled clinical trial. | 0 | Ruxolitinib and MSCs. | MSCs and Ruxolitinib. | **Primary**: safety  **Secondary**: not informed | 70 (35 control group) | Routine treatment. | China |
| **ChiCTR2000030944 -** Randomized, open label, multi-center, controlled. | 1 | Natural Killer cells and MSCs as add-on therapy. | Natural Killer cells plus MSCs, dose and route not informed. | **Primary:** Changes of serum inflammatory factors  *Measure method: ELISA*  Patient death risk  Drug related adverse reactions and events. | 10 | Routine treatment. | China |
| **ChiCTR2000031139-** Interventional  Single-arm. | 0 | Human embryonic stem cell-derived M cells (CAStem cells). | Cell dose: 1x 3x10e6 cells / kg (IV), second infusion after 1 week, additional infusion possible. | **Primary:** Pulmonary function evaluation, Changes in blood gas analysis, Evaluation of activity, Evaluation of dyspnoea, Computed Tomography changes from baseline. | 20 | No | China |
| **ChiCTR2000029572 -** Randomized, Parallel assignment,  Controlled, single mask (patients). | 0 | UC-Mononuclear cells as add-on therapy | Intravenous | **Primary:** Pneumonia Severity Index (PSI). | 30 (15 each group) | Routine treatment | China |
| **ChiCTR2000029569 -**  Randomized, Parallel assignment,  Controlled, open label | 0 | UC-MSC-derived conditioned medium as add-on therapy | Not informed | **Primary:** Pneumonia Severity Index  **Secondary:** CT, x-ray, arterial blood gas, assistant breathing use time, mortality, disease evolution, hospitalization days, safety outcome index | 30 (15 each group) | Routine treatment | China |
| **ChiCTR2000030484 -** Interventional Randomised  Placebo-controlled | NA | UC-MSCs and UC-MSC-derived exosomes | HUMSCs: two doses of 5X 10^7^ cells, days 1 and 8, intravenous.  Exosomes: two doses of 180mg, days 1 and 8, intravenous. | **Primary:** Ratio of partial pressure arterial oxygen and fraction of inspired oxygen (PaO2/FiO2) or respiratory rate (without oxygen); Frequency of respiratory exacerbation; recovery time; Computed Tomography and X-ray of lung; Time for cough to become mild or absent;  Time for dyspnea to become mild or no dyspnea; Frequency of oxygen inhalation or non-invasive ventilation, frequency of mechanical ventilation; C-reactive protein / Procalcitonin / Serum amyloid A, and other cytokine levels; Frequency of serious adverse events. | 90 (30 in each group) | Placebo | China |
| **EudraCT 2019-002688-89 -** Randomized, Controlled, Double-blind, Placebo controlled. | 1/2 | AT-MSC pulsed with H2O2. | Single dose of 1-2X10⁶ cells. | **Primary:** Adverse events, Average stay in the ICU, SOFA index, Mechanical ventilation, adverse events.  **Secondary:** Daily pulmonary mechanics; Determination of lung damage; Days free of vasopressors. | 26 | Placebo | Spain |
| **EudraCT 2020-001364-29 -** Multicenter, Randomized, Controlled | 1/2 | AT-MSCs as add-on therapy. | 16X10⁷ or 32X10⁷ cells, intravenous. | **Primary:** Incidence of Adverse Events and Serious Adverse Events according to the common toxicity criteria scale (CTCAE); Reduction of the SARS-CoV-2 viral load by PCR on days 6 and 15; Mortality at day 15; Mortality at day 28; Proportion of patients in categories 5, 6 or 7 of the ordinal scale of 7 points on days 15 and 28 days; Proportion of patients needing rescue therapy (Tocilizumab, corticosteroids, or therapies under investigation in clinical trials); Time to get an improvement in a category since admission to the ordinal scale. | 26 | Routine treatment. | Spain |
| **EudraCT 2020-001450-22 -** Controlled, Randomized, Multicenter, Open label. | 2 | UC-MSCs. | 1X10⁶ cells, intravenous. | **Primary:** Mortality due to lung involvement due to SARS-CoV-2 virus infection at 28 days of treatment.  **Secondary:** Mortality due to lung involvement ( 14, and 28 days after treatment); Days without mechanical respirator and vasopressor treatment for 28 days from randomization; Percentage of patients alive without mechanical ventilation and vasopressors on day 28 after randomization; Cure at 15 and 28 days; Safety and tolerability; PCR, PCT, Ferritin, D-Dimer, hsTroponin; immune subpopulations; Inflammatory markers, including interleukins Th1, Th2 and Th17, the innate immunity receptor NLRP3 and the highly mobile protein HMGB1. | 106 | Routine treatment. | Spain |
| **EudraCT 2020-001682-36 -** Double-blind, Randomized, placebo-controlled. | 1/2 | BM-MSCs. | 1X10⁷ cells, intravenous. | **Primary:** Proportion of patients in whom removal of invasive mechanical ventilation has been achieved in less than 7 days after IMP administration; Proportion of patients surviving on day 28 from diagnosis.  **Secondary:** Proportion of patients who have achieved a complete clinical response, with disappearance of the symptoms of the disease; Proportion of patients who have achieved a complete radiological response; Proportion of patients who did not require immunosuppressive treatment after treatment. | 24 | Placebo | Spain |
| **EudraCT 2020-001266-11 -** Two-center, randomized, controlled, Single blind. | 2 | AT-MSCs. | 1X10⁷ cells, intravenous. | **Primary:** % survival (day 28); days to normal temperature; days to weaning from mechanical ventilation; number of patients who weaned from mechanical ventilation; oxygen therapy duration and % of patient; days in ICU; days of hospitalization; Oxygen saturation; Pa PaO2 / FiO2 ratio; Radiological pattern on Chest Radiography; SOFA score; Murray score; Hb, Hto, VCM, Leukocytes with leukocyte formula, platelets. procalcitonin; Glucose, Glycosylated Hb, urea, uric acid, creatinine, total bilirubin, direct bilirubin, sodium, potassium, calcium, chlorine, total protein, C-reactive protein, AST, ALT, GGT, Cl Cr and FG; prothrombin time (TP), activated partial thromboplastin time (APTT), thrombin time (TT), Fibrinogen, INR; IL-6, IL-2, DD, lactate dehydrogenase, CK (Creatin Kinase), Alkaline Phosphatase; CD4 +, CD8 + T lymphocytes, B lymphocytes, NK cells or C3, C4, IgG, IgA, IgM. | 100 | Not informed. | Spain |
| **EudraCT 2020-001505-22 -** Double-blind, randomized, parallel, placebo-controlled. | 1/2 | WJ-MSCs. | 7-10X10⁵cells/kg, intravenous. | **Primary:** Number of patients who died on day +28, by treatment group  Secondary: Safety and feasibility; Number and percentage of patients who required rescue medication at day +28; Number of days of invasive mechanical ventilation up to day 28; survival free of mechanical ventilation; PaO2 / FiO2; SOFA Index; APACHE II score; Days of stay in the ICU; RT-PCR, LDH, D-dimer and Ferritin; percentage of leukocytes and neutrophils. | 30 | Placebo | Spain |

*Trial with published results

MSCs: Mesenchymal Stem Cells

AT-MSCs: Adipose Tissue Mesenchymal Stem Cells

EV-MSCs: Extracellular Vesicles derived from MSCs

OM-MSCs: Olfactory Mucosa Mesenchymal Stem Cells

PRP: Platelet-rich Plasma

WJ-MSCs: Wharton's Jelly Mesenchymal Stem Cells

UC-MSCs: Umbilical cord Mesenchymal Stem Cells

hESCs: human Embryonic Stem Cells
