# Supplementary material for: Mesenchymal Stem Cells: A New Piece in the Puzzle of COVID-19 Treatment
Source: Front Immunol. 2020 Jul 3;11:1563. doi: 10.3389/fimmu.2020.01563 (PMC7347794; doi:10.3389/fimmu.2020.01563)
Supplement: Supplementary file 2 [file Table_2.DOCX]

**Table 2.** Results of stem cell treatments for COVID-19.

| **Clinical trial identifier and characteristics** | **Intervention** | **Cell dose, route and other details** | **Results summary** | **Ref** |
| --- | --- | --- | --- | --- |
| **ChiCTR2000029990**, Interventional, Randomized, Parallel assignment | MSCs | Single dose of 1 × 10^6^ cells/kg, intravenous. | No adverse effects were observed. 7 out of 7 patients (100%) were cured or significantly benefited by MSC therapy. Severe and critically severe patients presented increased circulating Tregs,  as well as decreased overactivated T and NK cells. Treated patients also presented increased IL-10, IP-10, VEGF levels, and lower TNF-a production, compared to untreated patients. | (26) |
| **NCT04371393 -** Interventional, Randomized, Parallel Assignment, Triple Masking (Participant, Investigator, Outcomes Assessor) | Allogeneic Stem Cells (Remestemcel-L) | Two doses of 2 X10^6^ cells/kg, 4 days apart (± 1 day) | Survival of 10 out of 12 (83%) of COVID-19 patients with moderate/severe disease, of which 9 (75%) came off ventilator support after 10 days. | (80) |
| **Not informed A -** Randomized, Double-Blind, Placebo-Controlled Study | CAP-1002 (Allogeneic Cardiosphere-Derived Cells) as add-on therapy | Maximum of two doses of 150 million cells, intravenous, 6 days apart (± 1 day) | No adverse effects were observed. 4 out of 6 (66.6%) of patients came off ventilator support and discharged. | (81) |
| **Not informed B -** Non-randomized, prospective, open-label | ExoFlo - BM-MSC-derived exosomes as add-on therapy | Single dose of ExoFlo, intravenous. | No adverse effects attributable to treatment were observed. Overall survival rate of 20 out of 24 (83%) patients. 17 out of 24 (71%) patients were discharged after approximately 5.6 days of treatment. 3 out of 24 (13%) patients remained critically ill after treatment. | (84) |
| **Not informed C -** Non-randomized, prospective, single group, open label | WJ-MSCs | Single dose of 1X10^6^ cells/kg, intravenous | The pulmonary function and symptoms of the single patient investigated improved after hWJC transplantation. Patient was discharged 7 days after treatment. The percentage and counts of lymphocyte subsets (CD3+, CD4+, and CD8+ T cell) and IL-6, TNF-α, and C-reactive protein tended to normalize following treatment. | (82) |
| **Not informed D** | UC-MSCs | Three doses of 5X10^7^ cells at days 0, 3 and 6 | No adverse effects were observed. The unique patient treated in the study presented improved biochemical (serum bilirubin, CRP, and ALT/AST), immunological (white cell count, neutrophil count, CD3+ T cell, CD4+ T cell, and CD8+ T) and clinical parameters (pneumonia and ICU leave). | (83) |

MSCs: Mesenchymal Stem Cells

BM-MSCs: Bone Marrow Mesenchymal Stem Cells

WJ-MSCs: Wharton's Jelly Mesenchymal Stem Cells

UC-MSCs: Umbilical cord Mesenchymal Stem Cells
